# Late enhancement in 39 cardiac transplant patients: prevalence, pattern, and extent

**DOI:** 10.1186/1532-429X-11-S1-O21

**Published:** 2009-01-28

**Authors:** Craig RL Butler, Andreas Kumar, Mustafa Toma, Richard Thompson, Matthias Friedrich, David Ian Paterson

**Affiliations:** 1 University of Alberta, Edmonton, AB Canada; 2 University of Calgary, Calgary, AB Canada; 3 Stephenson Cardiovascular MR Center, Calgary, AB Canada

**Keywords:** Cardiovascular Magnetic Resonance, Myocardial Segment, Delay Enhancement, Inversion Recovery Sequence, Phase Sensitive Inversion Recovery

## Aim

To better characterize delayed enhancement patterns in the cardiac transplant population.

## Background

Cardiac transplant patients experience significant morbidity related to transplant vasculopathy and acute transplant rejection, both of which can cause scarring of the myocardium. Contrast enhanced cardiovascular magnetic resonance (CMR) has the unique ability to visualize and quantify myocardial scarring. It is well understood that myocardial infarctions resulting from transplant vasculopathy adversely affect prognosis and modify therapy. There is a growing body of evidence from non-transplant disease states, that the presence of non-infarct myocardial scar is also correlated to poor prognosis. Currently there is very little data on the scarring patterns present in the cardiac transplant population and it is our goal to better describe this pathology.

## Methods

Thirty-nine transplant patients underwent contrast enhancement imaging at the time of routine myocardial biopsy at two hospital centers in Alberta, Canada. Standard phase sensitive inversion recovery sequences were used on commercially available scanners (Siemens Avanto and Sonata, Siemens, Erlangen, Germany). Delayed enhancement (DE) was evaluated visually using CMR^42^ (Circle Canada Inc, Calgary, Canada) software analysis package by two independent readers. DE had to be cross-referenced in two orthogonal views. Disagreements were settled by consensus. The extent of DE was assessed semi-quantitatively by scoring each of the 17 myocardial segments according to the proportion of DE in each segment (1 = 75%). The scores of the 17 individual myocardial segments were added together to give an aggregate DE burden.

## Results

**Figure 1:**
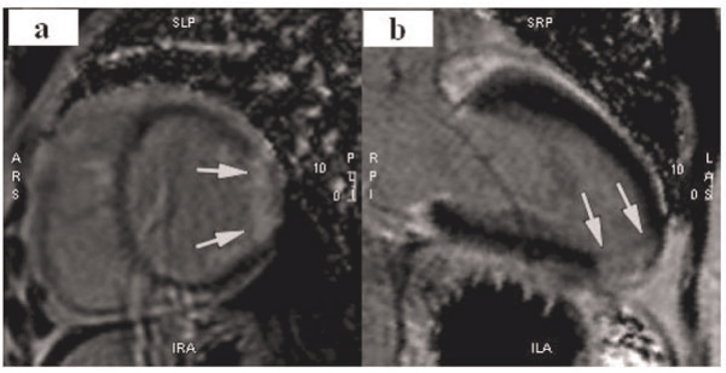
**Transmural lateral wall infarction (a) and Inferoapical infarction (b)**.

**Figure 2:**
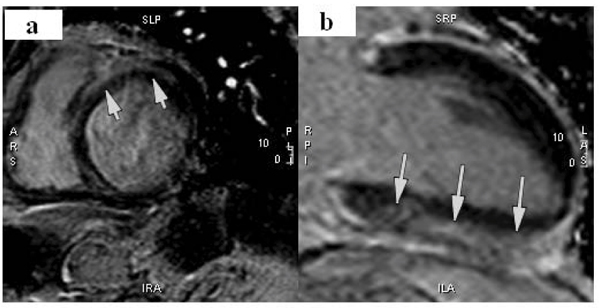
**Example of non-ischemic fibrosis**. Subepicardial delayed enhancement of the anteroseptal and anterior walls (a) and inferior wall (b).

**Figure 3:**
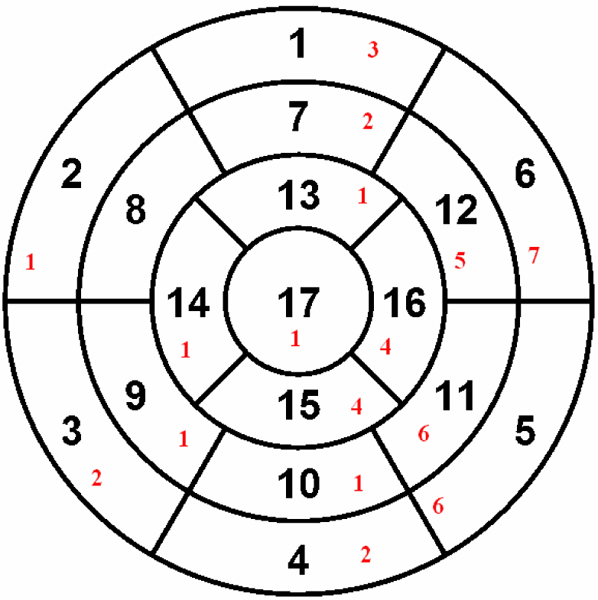
**Frequency of any delayed enhancement by myocardial segment**.

Three (8%) out of 39 patients scanned had to be excluded due to poor image quality. There were seven women (18%) and thirty two men (82%). Fifteen (45%) patients had grade 1R cellular rejection rejection, and two (6%) had grade 2R rejection. Mean time since transplant was 37 months (standard deviation = 55 months). Eighteen (50%) of 36 patients had DE. Among patients with DE, four patients (22%) had a subendocardial or transmural pattern consistent with myocardial infarction (Figure 1), and 14 (78%) had a midwall or subepicardial pattern (Figure 2) consistent with non-ischemic injury. Overall, patients with DE had scores ranging from 1 to 19, with a mean of 5.4 (standard deviation = 4.8). Non-ischemic DE was most commonly seen in the anterolateral and inferior walls (Figure 3). There was no significant association between the presence of DE and time since transplant or current biopsy result.

## Conclusion

DE is a common feature in the transplant population. Most DE observed is in a non-ischemic pattern; however a significant proportion had DE patterns consistent with infarction. The relationship between DE and cumulative episodes of rejection, hospitalization, and long term prognosis needs to be explored in more detail.

